# Ideal placement of an implant considering the positional relationship to an opposing tooth in the first molar region: a three-dimensional finite element analysis

**DOI:** 10.1186/s40729-020-00223-9

**Published:** 2020-07-01

**Authors:** Jun Morita, Masahiro Wada, Tomoaki Mameno, Yoshinobu Maeda, Kazunori Ikebe

**Affiliations:** 1 Private Dental Office, Moriyama, Shiga Japan; 2 Department of Prosthodontics, Gerodontology and Oral Rehabilitation, Osaka University Graduate School of Dentistry, 1-8, Yamadaoka, Suita, Osaka, 565-0871 Japan

**Keywords:** CBCT, Dental implants, Finite element analysis, Stress distribution

## Abstract

**Background:**

Excessive loading from the occlusion is known as a major pathological factor in implant failure. The force applied to the implant varies depending on the positional relationship to an opposing tooth in clinical cases. However, no studies have clarified the relationship between the discrepancy and mechanical complications.

**Materials and methods:**

The study enrolled patients whose mandibular first molar was missing and was opposed by a natural maxillary first molar. The horizontal and vertical distance between the residual ridge and the occlusal surface of the maxillary first molar were measured from computerized tomograms. Subsequently, four finite element models were constructed in combinations of horizontal and vertical discrepancies. Additionally, the effect of inclined implantation and angled abutments were examined in a large clearance model. Maximum von Mises stress values generated in abutments under 90° or 60° loading vectors were compared with a three-dimensional finite element method.

**Results:**

Data from 123 subjects (39 males and 84 females, average age 55.2 ± 11.4 (SD) years) were collected for the analyses. Under all conditions, the stress on the load side (the buccal side) was concentrated on the platform, and the stress on the opposite side (the lingual side) was concentrated on the top of the abutment tube inserted into the implant. In comparison to 90° loading vectors, the maximum von Mises stresses of each model were 1.20 to 2.67 times under 60° loading vectors. For inclined implantation, the maximum stress was 8.4% less at a 90° load and 9.7% less at a 60° load compared with vertical implantation. With angled abutments, the maximum stress was 15.7% less at a 90° load and 30.0% less at a 60° load compared with vertical implantation.

**Conclusion:**

In cases of progressive alveolar resorption with a large clearance between the implant and the opposing teeth, a higher stress concentration was observed at the joint between the implant and the abutment. Our findings also showed that stress concentration around this area can be reduced by the use of inclined implantation and angled abutments under the condition of a horizontal offset between the implant and opposing teeth.

## Background

Alveolar bone resorption inevitably occurs after a tooth is extracted. The residual ridge takes various forms after extraction depending on the degree of inflammation before tooth extraction, the thickness and quality of the bone surrounding the extraction cavity, and bone damage caused by the extraction. This diversity [1, 2] results in differences in the buccolingual position and the vertical distance between the alveolar ridge and the opposing teeth.

In recent decades, implant-supported prostheses have been increasingly used, and the survival rate of implants is reported to be almost 100% over the course of 5 years as a result of advances in the implant material and shape, surface characteristics, and surgical protocols [3–5]. In other words, failure of the implant itself is becoming extremely rare if the patient’s treatment takes into account systemic/local conditions, the use of appropriate materials and surgical procedures, and the completion of regular maintenance. However, it has been reported that mechanical complications of prostheses (such as loosening or fracture of abutment screws or the abutment itself, or tipping or fracturing of the facing material) increase with long-term use. According to systematic reviews [6, 7], the mechanical complications of a single-tooth implant prosthesis after 5 years is over 25%. Therefore, the long-term stability of the superstructure is also important for the success of implant treatment.

Esposito et al. [8] reported that excessive loading from the occlusion was a major pathological factor in implant failure. When there is a large discrepancy in the buccolingual position and/or the vertical distance between the alveolar ridge and the opposing tooth, an implant prosthesis is thought to be subjected to a larger occlusal load. This is because the further from the implant axis the loading vector is, the more the bending moment increases [9]. However, no studies have clarified the relationship between the discrepancy and mechanical complications in clinical cases.

The purpose of this study was to investigate the mechanical stress around implants under various conditions of buccolingual discrepancy and vertical distance between the residual ridge and opposing teeth in clinical cases. The ideal placement of an implant was also examined in consideration of the positional relationship to an opposing tooth using finite element model (FEM) analysis.

## Materials and methods

### Participants

This study focused on the mandibular first molar, which is the most frequent location for a single-tooth implant. The study population consisted of patients whose mandibular first molar was missing and was opposed by a natural maxillary first molar, and who received implant treatment at a private dental office from June 2007 to August 2009. Patients who had an opposing tooth with severe periodontal disease or marked dislocation out of the dentition were excluded from the analyses. Before implant placement, all patients underwent cone-beam computerized tomography (CBCT) using a 3DX multi-image micro CT FDP (Morita Co., Kyoto, Japan) with a tube current time of 52.5 mAs, a tube voltage of 80 kVp, and the occlusal plane of the patient parallel to the floor surface. This study protocol was approved by the Osaka University Graduate School of Dentistry Ethics Committee (H21-E8). Every clinical investigation was conducted according to the principles expressed in the Helsinki Declaration.

### Experiment 1

The aim of experiment 1 was to evaluate a variety of buccolingual discrepancies and vertical distances between the residual ridge and the opposing teeth in clinical cases using diagnostic imaging for implant treatment.

**Fig. 1:**
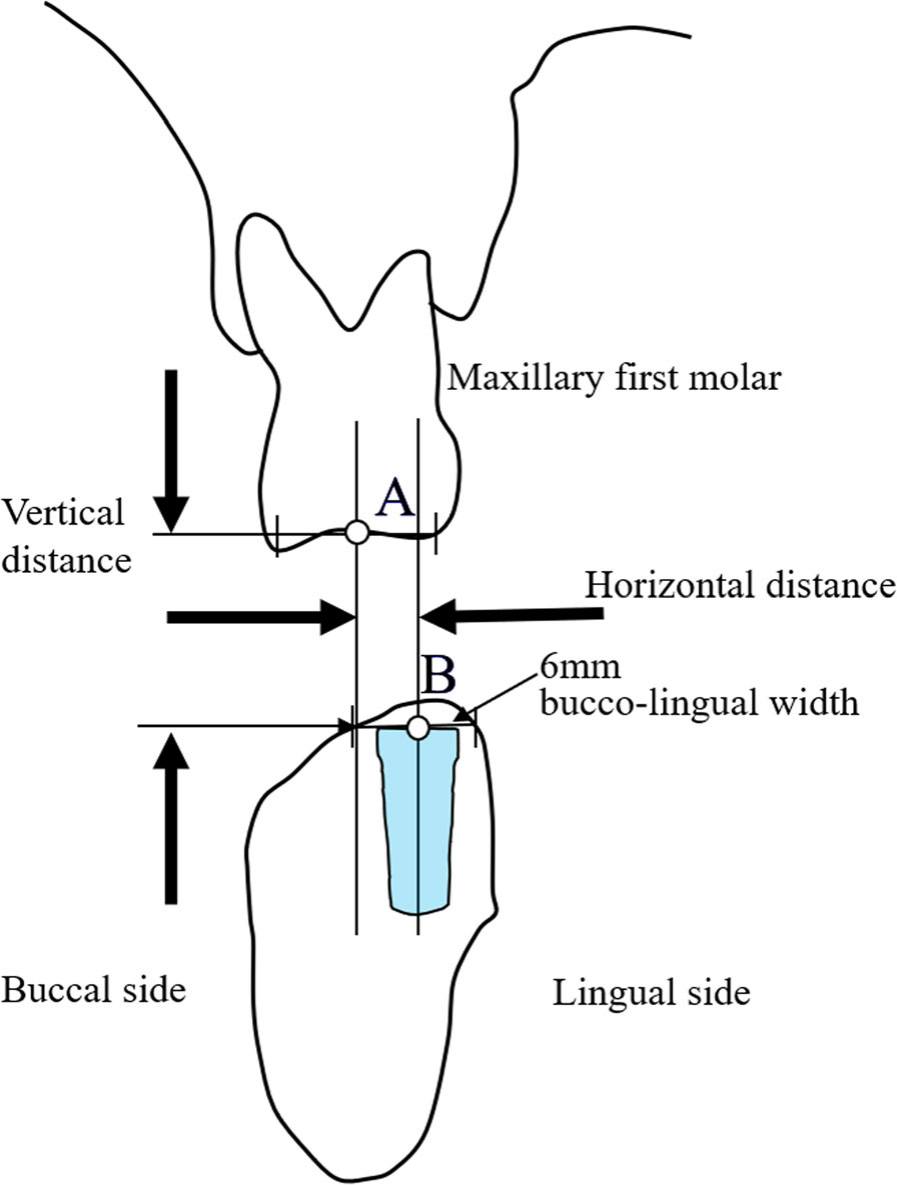
Cross-sectional image perpendicular to the dental arch passing through the mesiodistal midpoint of the maxillary first molar: an implant in the mandible first molar region. A: reference point of the maxillary first molar: the buccopalatal midpoint of the occlusal surface of the maxillary first molar. B: reference point of the maxillary first molar: buccolingual midpoint of the occlusal side of the implant

A cross-sectional CBCT image perpendicular to the dental arch passing through the mesiodistal midpoint of the maxillary first molar was used for measurements. The horizontal and vertical distance between the residual ridge in the mandibular first molar region and the occlusal surface of the maxillary first molar were measured (Fig. 1). For the measurement, the reference point of the maxillary first molar (point A) was defined as the buccopalatal midpoint of the occlusal surface. The bone width around the implant neck required at least 6 mm to preserve 1 mm of intact bone when using a 4-mm diameter implant. Point B was defined as the buccolingual midpoint of 6 mm bone width measured at the occlusal side using the coronal CBCT image. Then, the horizontal and vertical distance between points A and B were measured.

**Fig. 2:**
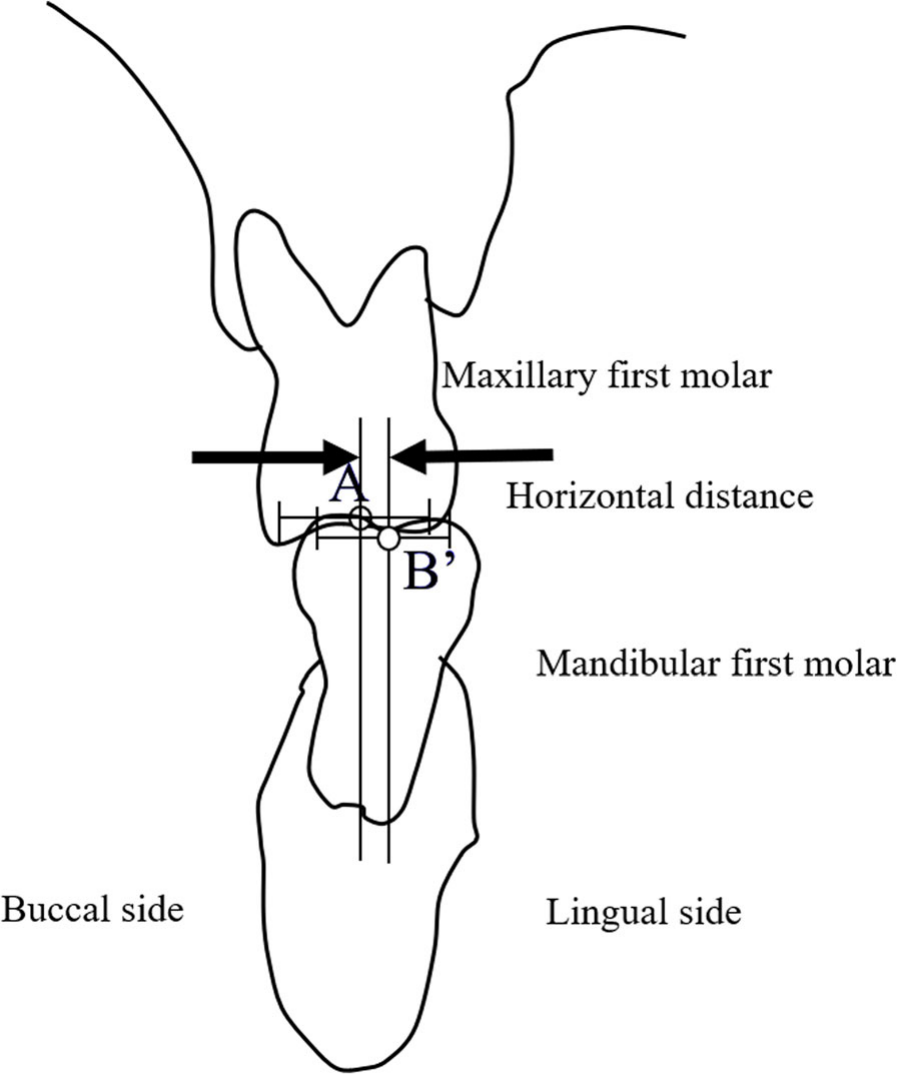
Cross-sectional image perpendicular to the dental arch passing through the mesiodistal midpoint of the maxillary first molar: a natural mandibular first molar. A: reference point of the maxillary first molar. B’: reference point of the mandibular first molar: buccolingual midpoint of the occlusal surface of the mandibular first molar

As a control, patients with natural opposing maxillary and mandibular first molars were selected. The horizontal discrepancy between the buccolingual center of the occlusal surface of the maxillary (point A) and mandibular (point B’) first molars was also measured (Fig. 2).

At first, the histograms of the horizontal discrepancy (A–B, A–B’) and the vertical distance (A–B) were examined. Then, the horizontal distance between the maxillary first molar and the mandibular residual ridge (A–B) was compared to the distance between the maxillary and mandibular first molars (A–B’) by paired *t* test. Next, associations of the horizontal discrepancy between the maxillary first molar and the mandibular residual ridge (A–B) with sex and age were evaluated by *t* test and Pearson’s correlation coefficient test.

### Experiment 2

The aim of experiment 2 was to analyze the stress distribution on dental implants and abutments with the axis perpendicular to the occlusal plane using three-dimensional (3D) FEMs with various conditions of horizontal and vertical distances.

#### 3D finite element models

**Fig. 3:**
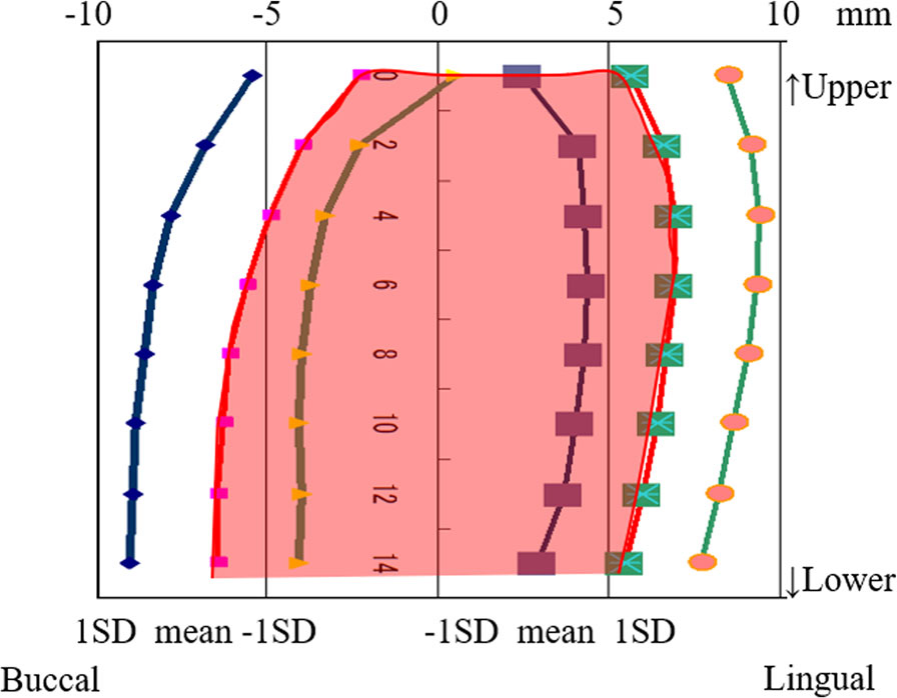
The average shape of the mandibular bone in the first molar region

**Fig. 4:**
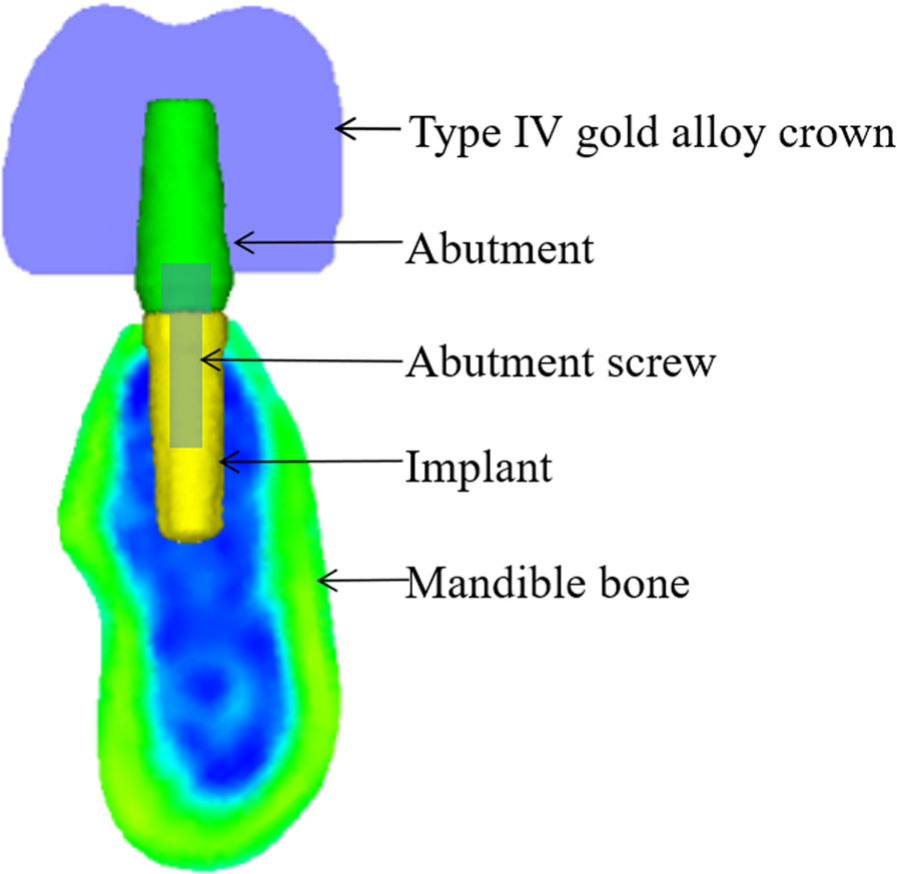
3D finite element models

**Fig. 5:**
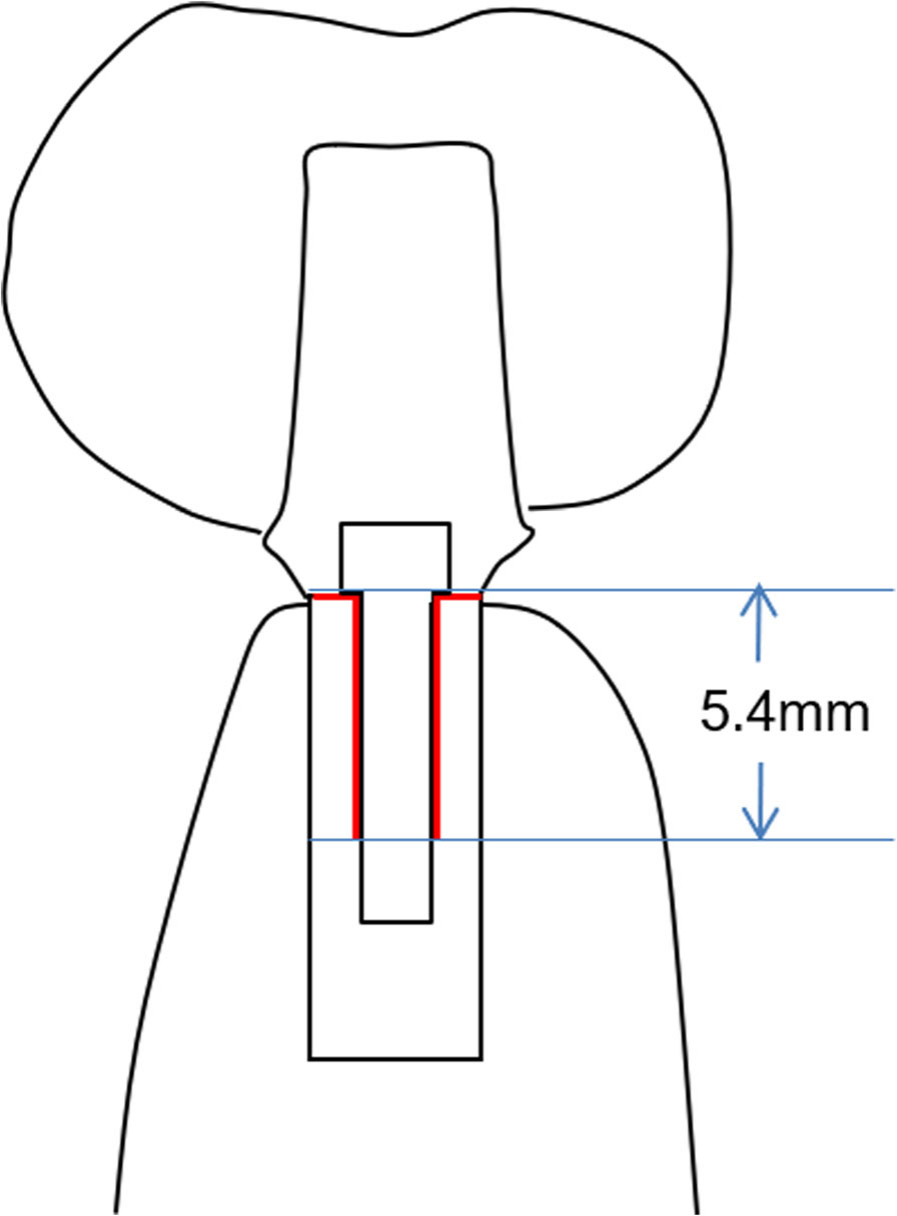
The range of the slip boundary condition

The buccal and lingual shape of the mandibular bone was measured every 2 mm on a vertical line according to the method of experiment 1 (Fig. 3). Subsequently, the average shape of the mandibular bone in the first molar region was determined. Next, a patient (female, aged 53 years) whose mandibular bone was of the average shape was recruited. After explaining the purpose of this study and obtaining her consent, a computed tomography (CT) scan was taken using a helical CT scanner (SOMATOM Definition Flash AS, SIMENS Co., Berlin, Germany) with a tube current time of 270 mAs and a tube voltage of 120 kVp. Similarly, a bone level implant with an internal connection (SCREW-LINE implant, 3.8 mm diameter, 11 mm length, CAMLOG Biotechnologies ALTATEC GmbH, Basel, Switzerland), an abutment (Standard abutment, straight, 3.8 mm diameter, GH 1.5, CAMLOG Biotechnologies ALTATEC GmbH, Basel, Switzerland), and a type IV gold alloy crown were imaged using a helical CT scanner. A 3D FEM was constructed from the CT scan images using a computer program (Mechanical Finder Version 6.0, Research Center of Computational Mechanics Inc., Tokyo, Japan) (Fig. 4). The FEM was set under the following characteristics:

- The implant was inserted perpendicular to the occlusal plane.
- The abutment was fixed to the implant with an abutment screw.
- The crown was cemented to the abutment with no cement layer.
- Computational fluid mechanics between the implant and abutment were set to a slip boundary condition for understanding the mechanical behavior between implant and abutment junction. Meanwhile, those of the others (between the mandibular bone and the implant, the implant and the abutment screw, the abutment screw and the abutment, and the abutment and the crown) were set to a no-slip boundary condition (Fig. 5).

**Fig. 6:**
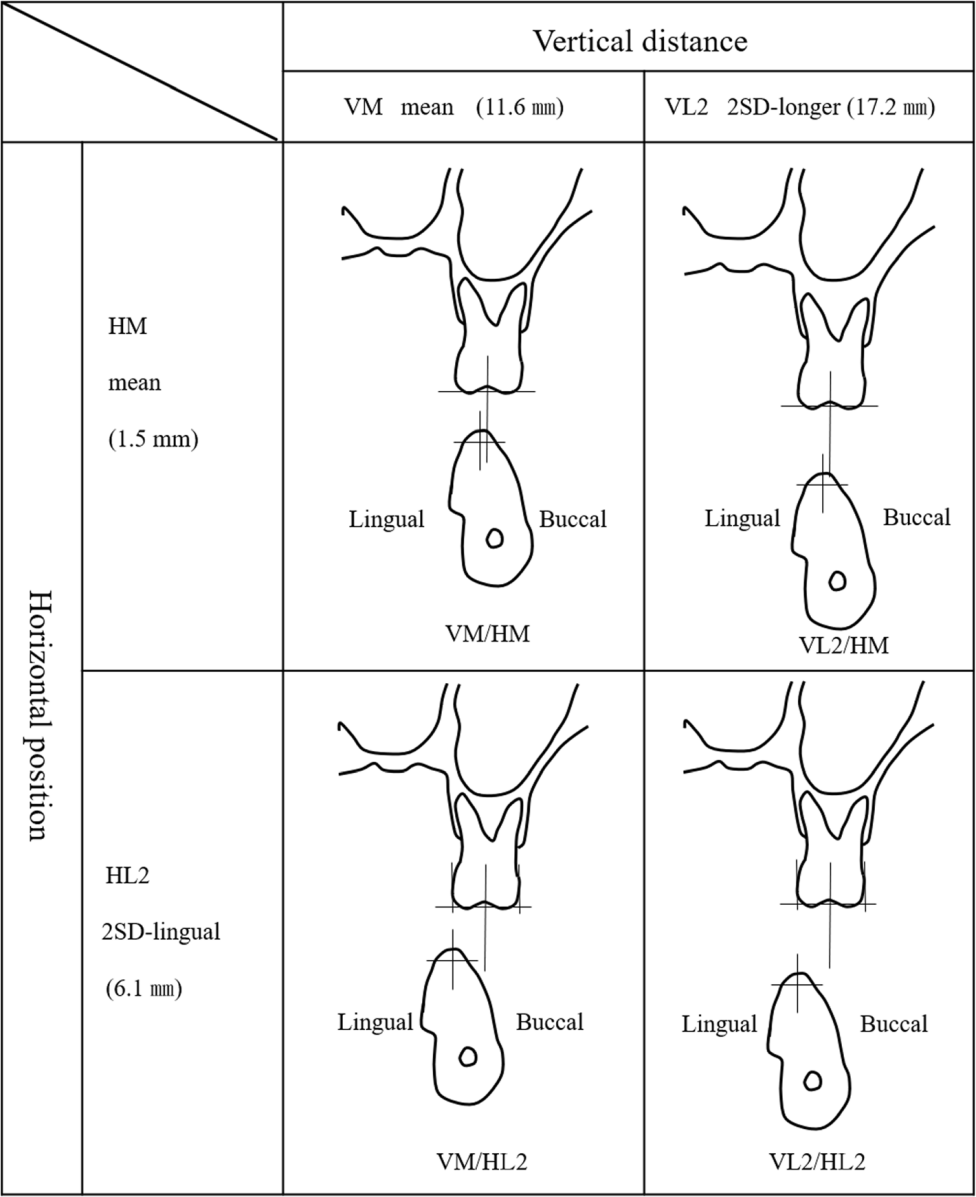
The horizontal and vertical position between the maxillary first molar and the mandibular residual ridge

In total, four FEMs were constructed in combinations of horizontal (HM or HL2) and vertical (VM or VL2) discrepancies between points A and B (Fig. 6). HM had an average horizontal position (point B was located 1.5 mm lingual to point A) and HL2 had a 2SD-lingual (6.1 mm) horizontal position. Likewise, the average vertical distance between points A and B was 11.6 mm (VM) and the 2SD vertical distance was 17.2 mm (VL2).

#### Constraint and loading conditions

**Fig. 7:**
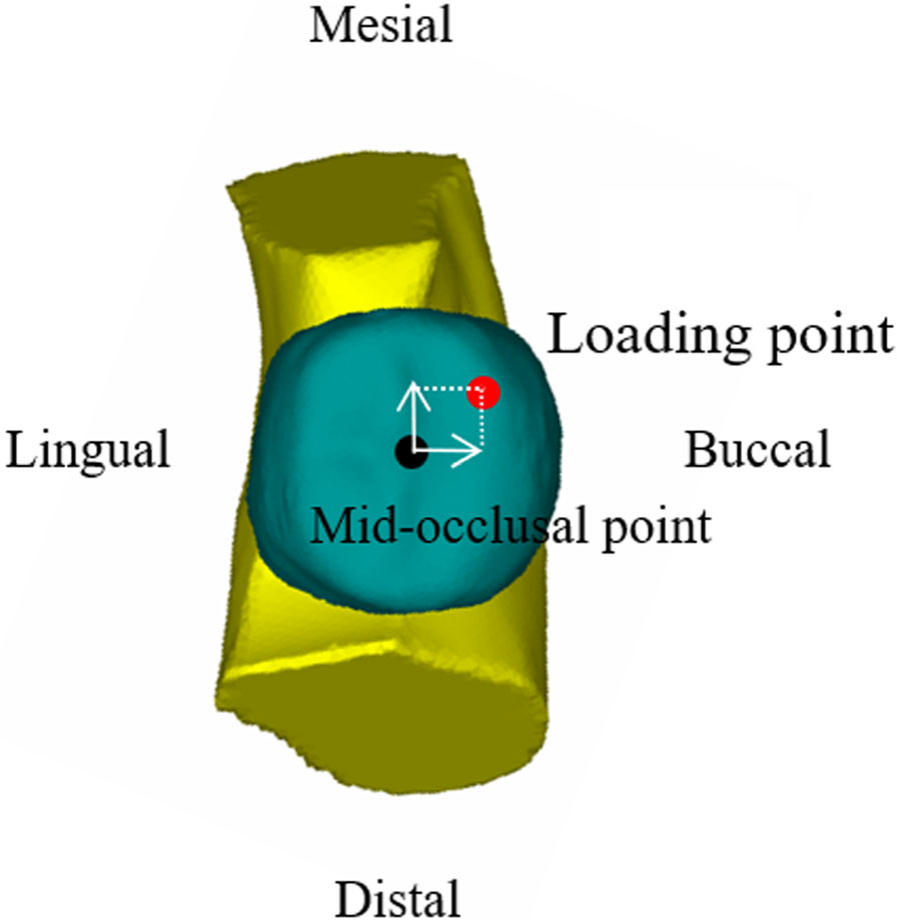
Loading conditions

**Fig. 8:**
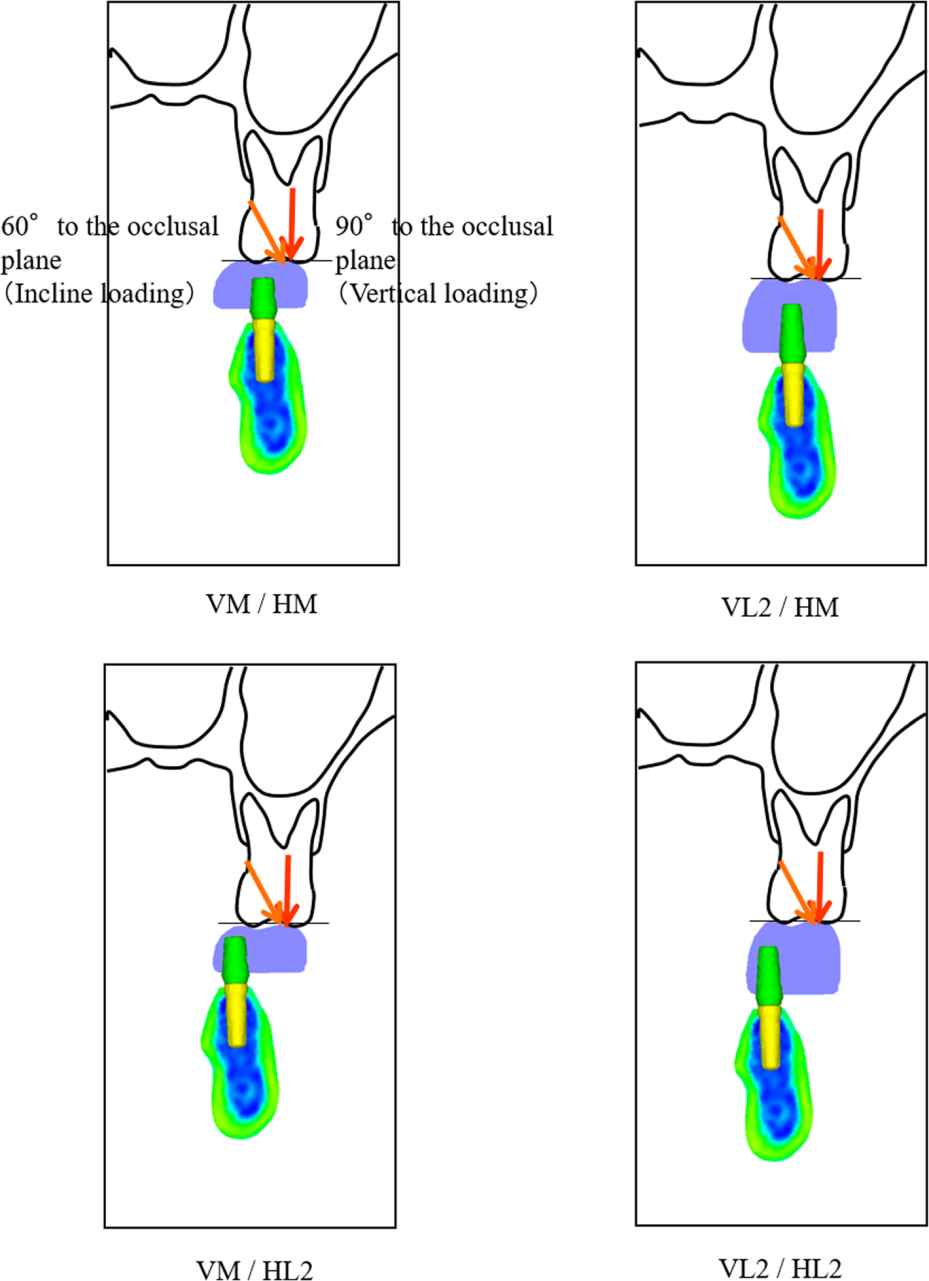
Positional relationship and loading vectors

Movement was restricted on the inferior border of the mandible. A loading point was set at the mesiobuccal cusp (2 mm buccal and 2 mm mesial from point A) (Fig. 7). A 120 N compression axial force was applied to simulate the maximum force generated on the first molar by the normal mastication process. The loading vectors were simulated with angles of 90° and 60° to the occlusal plane (Fig. 8).

#### Mesh generation and material properties

The mesh was generated with tetrahedral quadratic elements. The number of quadrilateral elements of the implant, the abutment, and the abutment screw were 52085–52414, 51325–59546, and 26055–26242, respectively. Material properties for each unit were set according to previous studies except for bone, which was set using the Keyak formula [10] (Table 1).

**Table 1:** Material properties for each unit

Component	Material	Young modulus [Mpa]	Poisson ratio
Implant	Titanium	110,000	0.35
Abutment	Titanium	110,000	0.35
Abutment screw	Titanium	110,000	0.35
Crown	Type IV gold alloy	90,000	0.3
Residual ridge		*	0.4

*The Young modulus was set according to Keyak formula

#### Data analysis

The results of the analysis were produced numerically and converted to visual results with color codes. Maximum von Mises stress values generated in abutments were compared.

### Experiment 3

**Fig. 9:**
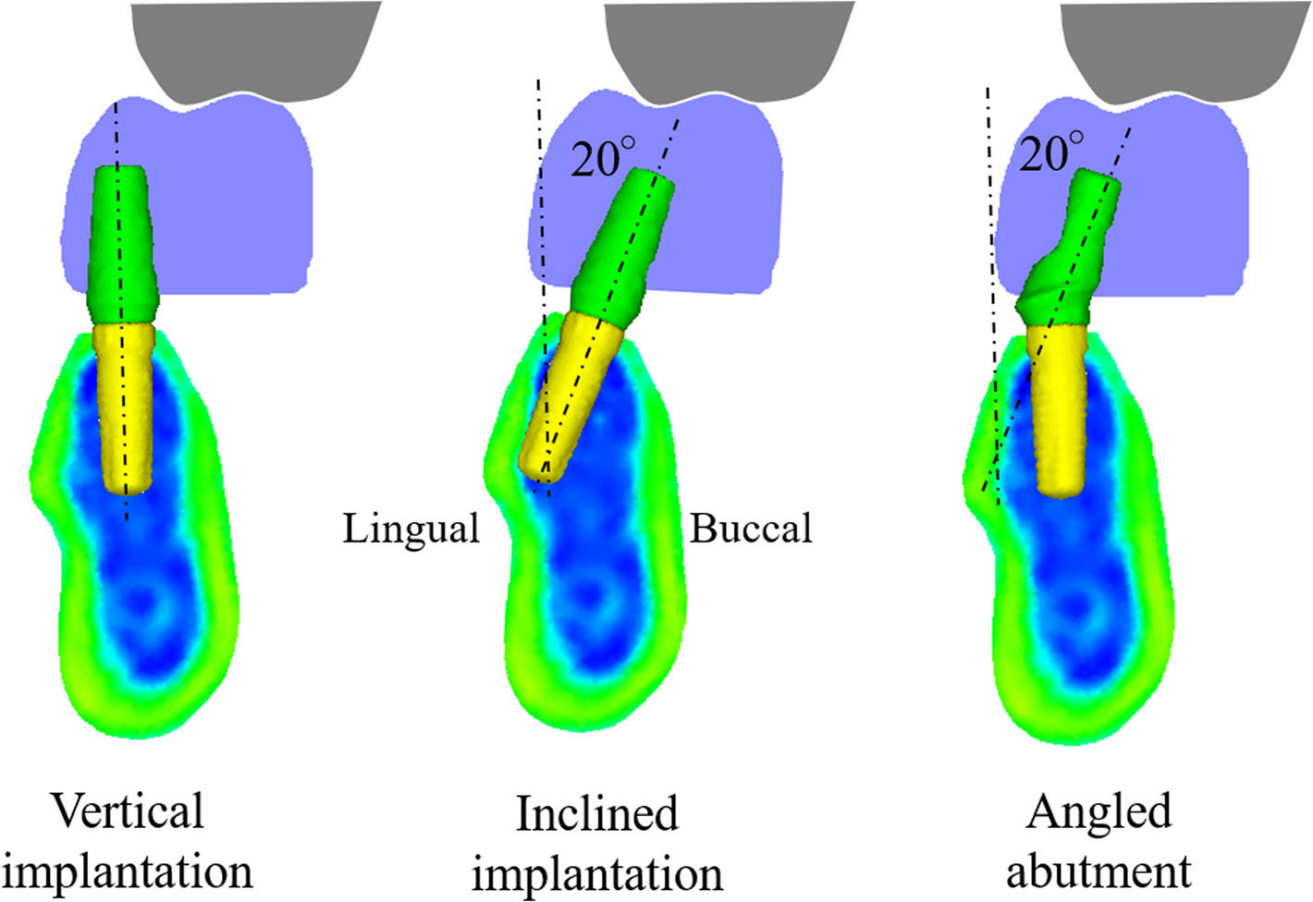
Three models under conditions of HL2/VL2

The aim of experiment 3 was to examine the effect of inclined implantation and angled abutments. Three models were created in which the implant was inserted vertical to the occlusal plane (vertical implantation model), in which the implant was inserted at a 20° inclination to the buccal side (inclined implantation model), and in which the implant was inserted vertically and used a 20° angled abutment (angled abutment model) under the conditions of HL2/VL2 (Fig. 9). The shape and position of the crown of the teeth were the same.

Constraint conditions, load conditions, elements, and material constants were the same as in experiment 2. Mises equivalent stress generated in the abutment was compared with a 3D finite element method.

## Results

### Experiment 1

Data from 123 subjects (39 males and 84 females, average age 55.2 ± 11.4 (SD) years) were collected for the analyses. Twenty-seven subjects were selected as a control group.

**Fig. 10:**
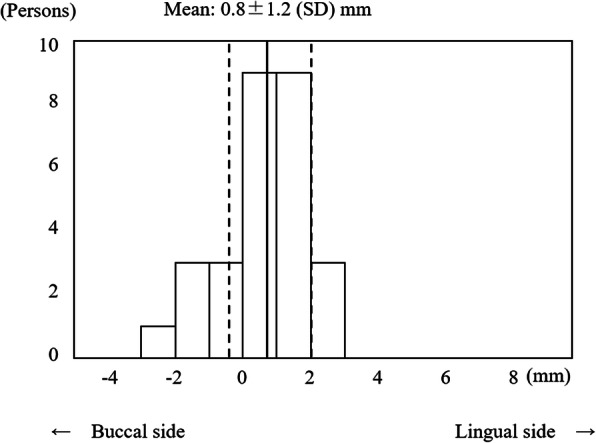
Distribution of the horizontal distance from the maxillary to mandibular first molars (A–B’) (*n* = 27)

**Fig. 11:**
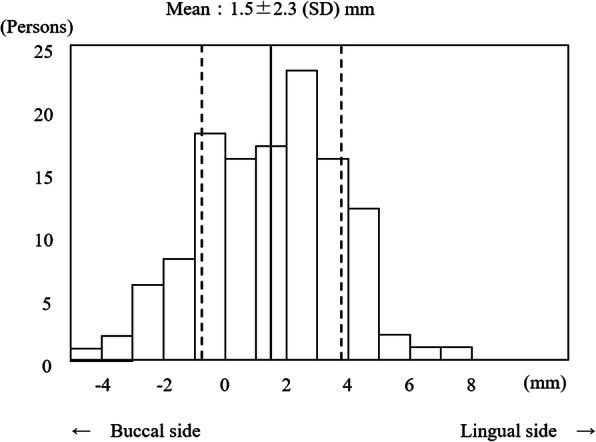
Distribution of the horizontal distance from the maxillary first molar to the mandibular residual ridge (A–B) (*n* = 123)

The buccolingual center of the occlusal surface of the mandibular first molar (point B’) was located 0.8 ± 1.2 (SD) mm to the lingual side of the maxillary first molar (point A) (Fig. 10). The buccolingual center of the residual ridge (point B) was located 1.5 ± 2.3 mm to the lingual side. Additionally, point B was more widely distributed (from 4.9 mm on the buccal side to 7.8 mm on the lingual side) (Fig. 11). The horizontal distance between the maxillary molar and the mandibular residual ridge (A–B) was significantly larger than that between the maxillary and mandibular molars (A–B’) of the same subjects (*p* = 0.025), and the mandibular residual ridge was located more to the lingual side. The distance between A and B was found to be 1.1 ± 2.6 mm for males (39 subjects) and 1.6 ± 2.1 mm for females (84 subjects), but this difference was not significant. A weak but significant positive correlation was observed between the horizontal distance (A–B) and age (*r* = 0.213, *p* = 0.018). The horizontal distance between A and B was larger (2.0 ± 2.6 mm) in subjects missing both neighboring teeth (the second premolar and the second molar) compared with subjects with missing second premolars or second molars (1.2 ± 2.3 mm). However, this difference was not significant (*p* = 0.058).

**Fig. 12:**
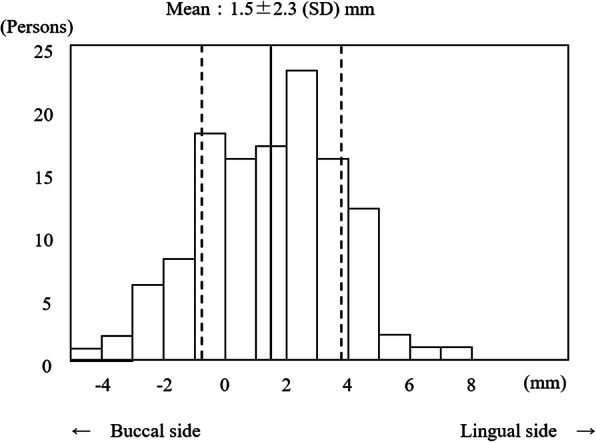
Distribution of the vertical distance between the maxillary first molar and the mandibular residual ridge (*n* = 123)

The vertical distance between the maxillary molar and the mandibular residual ridge (A–B) was widely distributed from 5.0 to 20.0 mm, and the average was 11.6 ± 2.8 mm (Fig. 12). The vertical distances were 12.2 ± 2.8 mm for males and 11.3 ± 2.7 mm for females (*p* = 0.068). There was also no significant relationship between age and the vertical distance (*r* = 0.090, *p* = 0.320). The vertical distance was greater (11.8 ± 2.6 mm) in subjects missing both neighboring teeth than in subjects with missing second premolars or second molars (11.5 ± 2.8 mm), but there was no significant difference (*p* = 0.559). There was also no significant correlation between horizontal distance and vertical distance between the maxillary molar and the mandibular residual ridge (*r* = - 0.029, *p* = 0.794).

### Experiment 2

#### Location of stress concentration

**Fig. 13:**
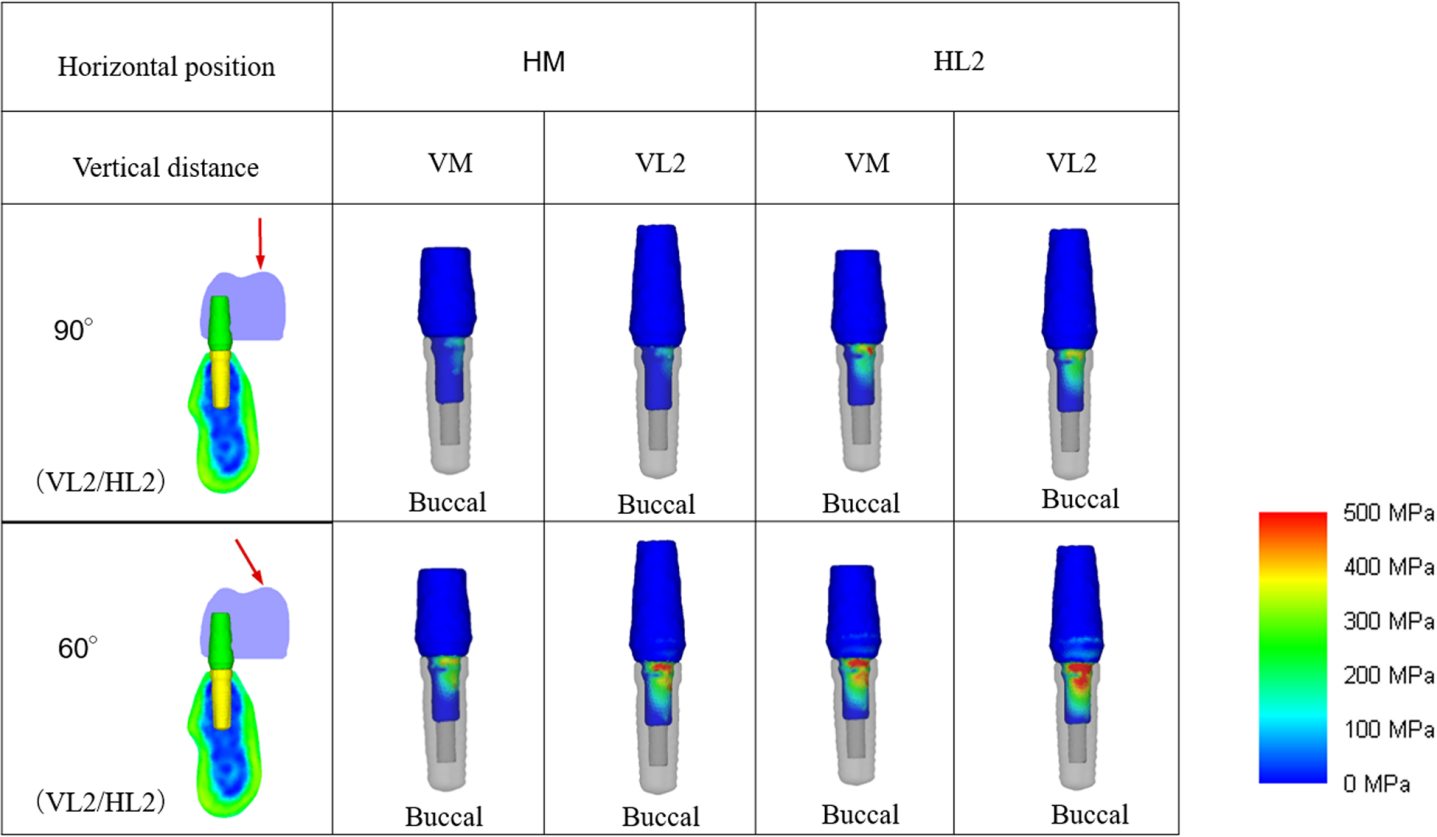
Distribution of the stress in the abutments of each model

Buccal stress was concentrated on the platform area, and lingual stress was concentrated at the top of the abutment tube area under all loading conditions. Distribution of the stress in the abutments of each model is illustrated in Fig. 13.

**Fig. 14:**
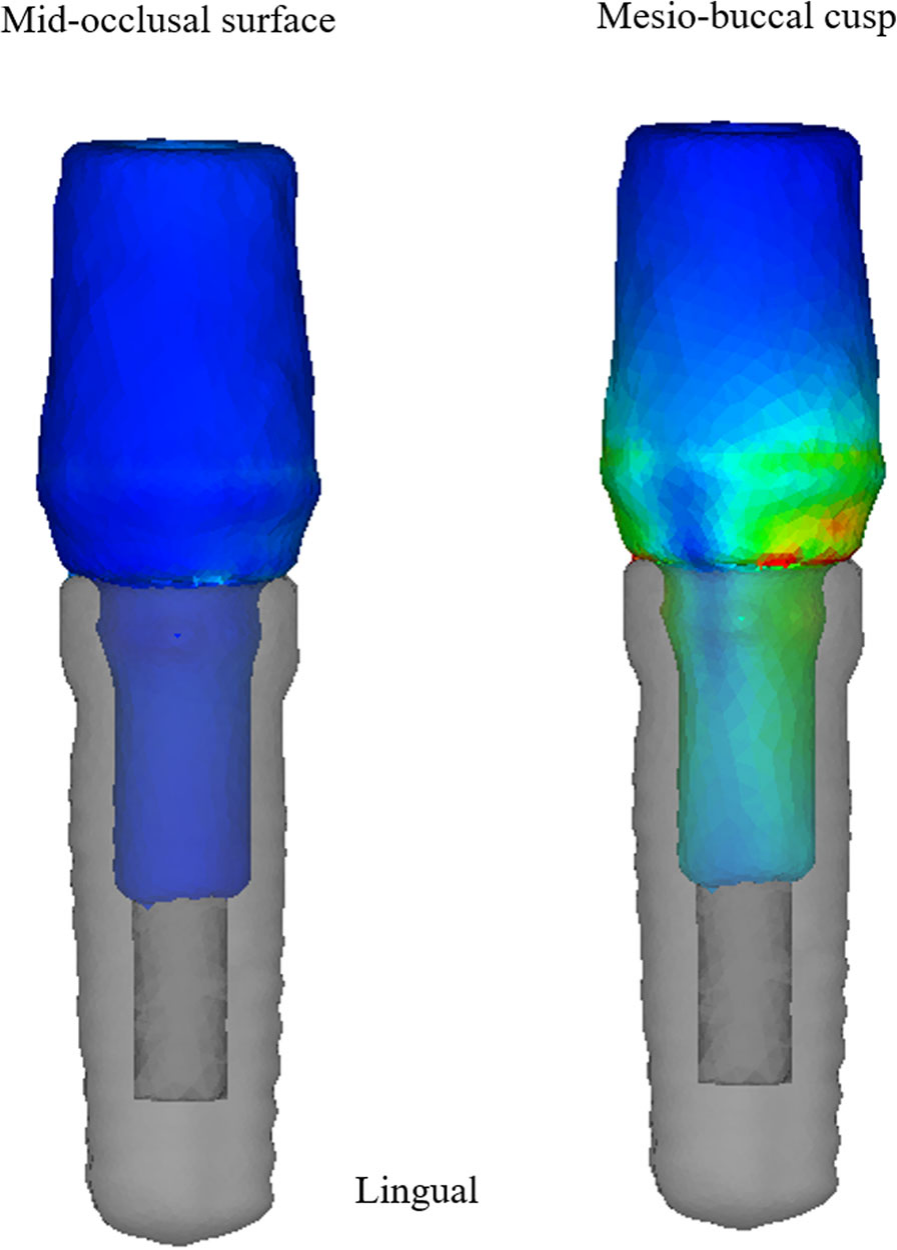
Distribution of the stress in the abutments of VM/HM under 90° loading at the mid-occlusal surface and the mesiobuccal cusp

Under all conditions, the stress on the load side (the buccal side) was concentrated on the platform, and the stress on the opposite side (the lingual side) was concentrated on the top of the abutment tube inserted into the implant. Additionally, when the load was 90° under the condition of VM/HM, no buccolingual stress concentration occurred, and the stress value was also low (Fig. 14).

#### Difference of stresses in vertical conditions

In comparison with VM/HM, the maximum von Mises stress of VL2/HM was 1.12 times under 90° loading vectors and was 1.34 times under 60° loading vectors.

#### Difference of stresses in horizontal conditions

**Fig. 15:**
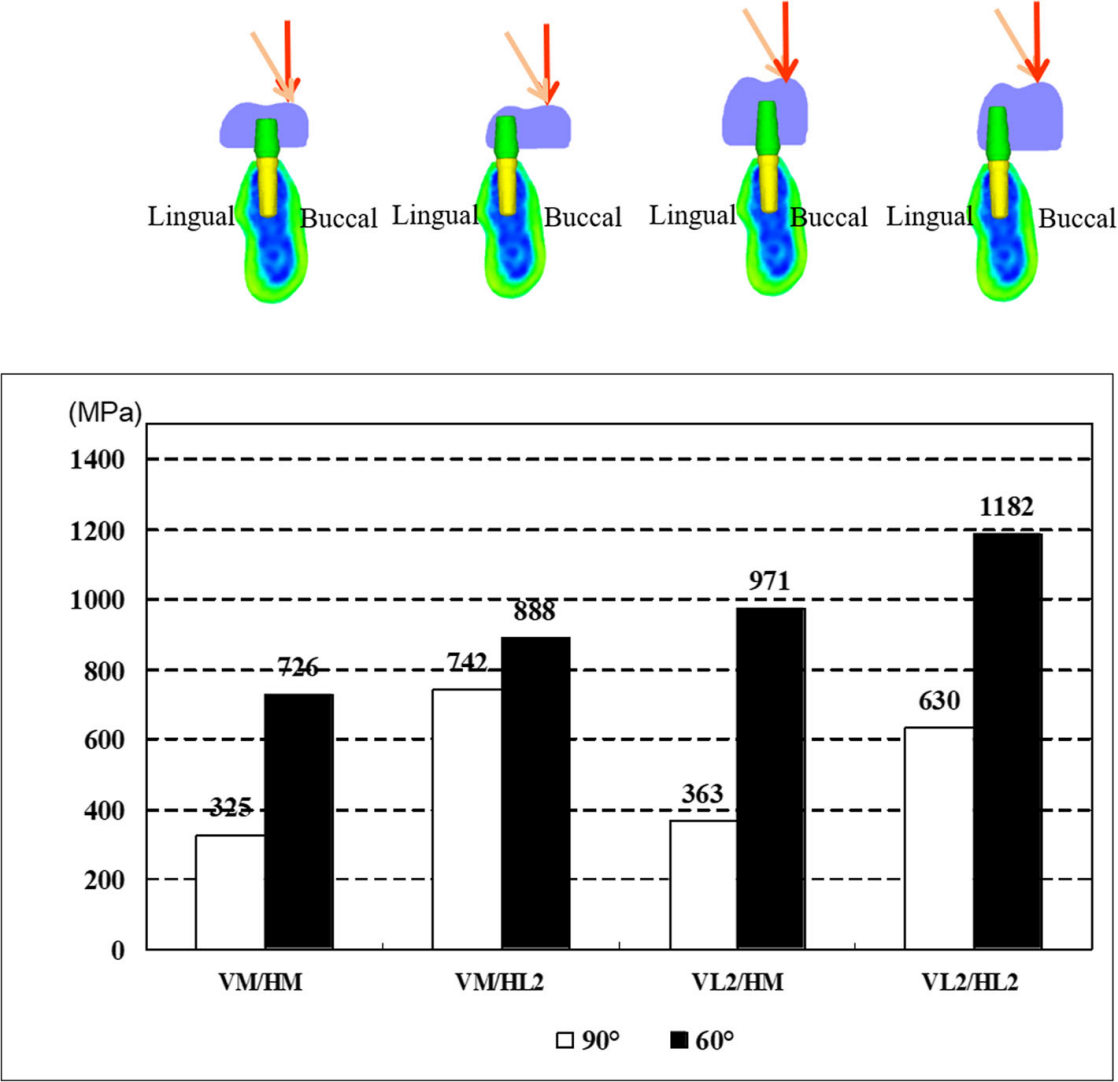
Comparison of the maximum stress in abutments under 90°or 60°loading

In comparison with VM/HM, the maximum von Mises stress of VM/HL2 was 2.37 times under 90° loading vectors and was 1.22 times under 60° loading vectors (Fig. 15).

#### Difference of stresses in loading conditions

In comparison to 90° loading vectors, the maximum von Mises stresses of each model were 1.20 to 2.67 times under 60° loading vectors (Fig. 15).

#### The condition inducing maximum stress

The maximum von Mises stresses were shown on VL2/HL2 under 60° loading vectors (Fig. 15).

### Experiment 3

#### Location of stress concentration

**Fig. 16:**
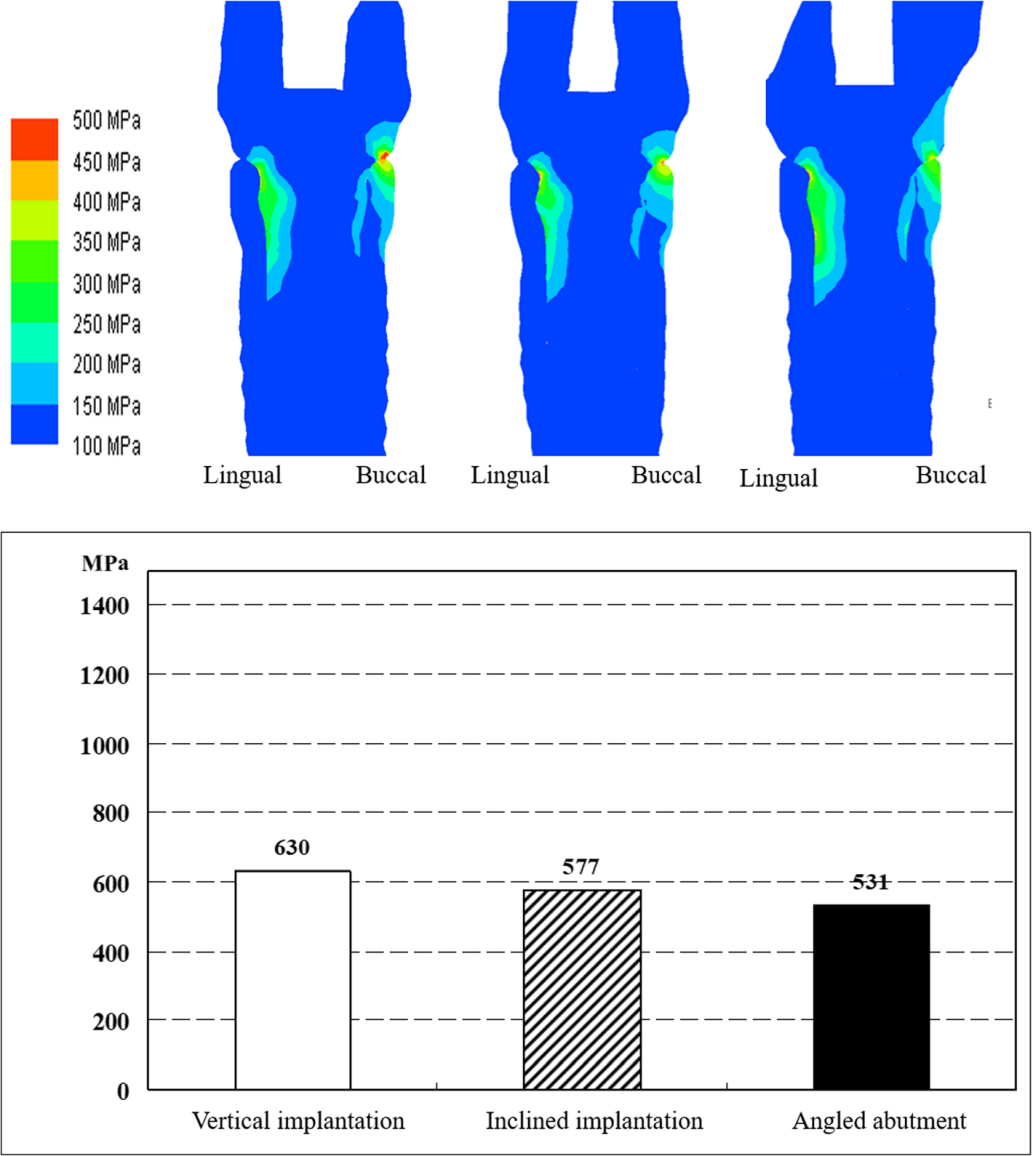
Comparison of the maximum stress under 90° loading in vertical implantation, inclined implantation, and angled abutment models

**Fig. 17:**
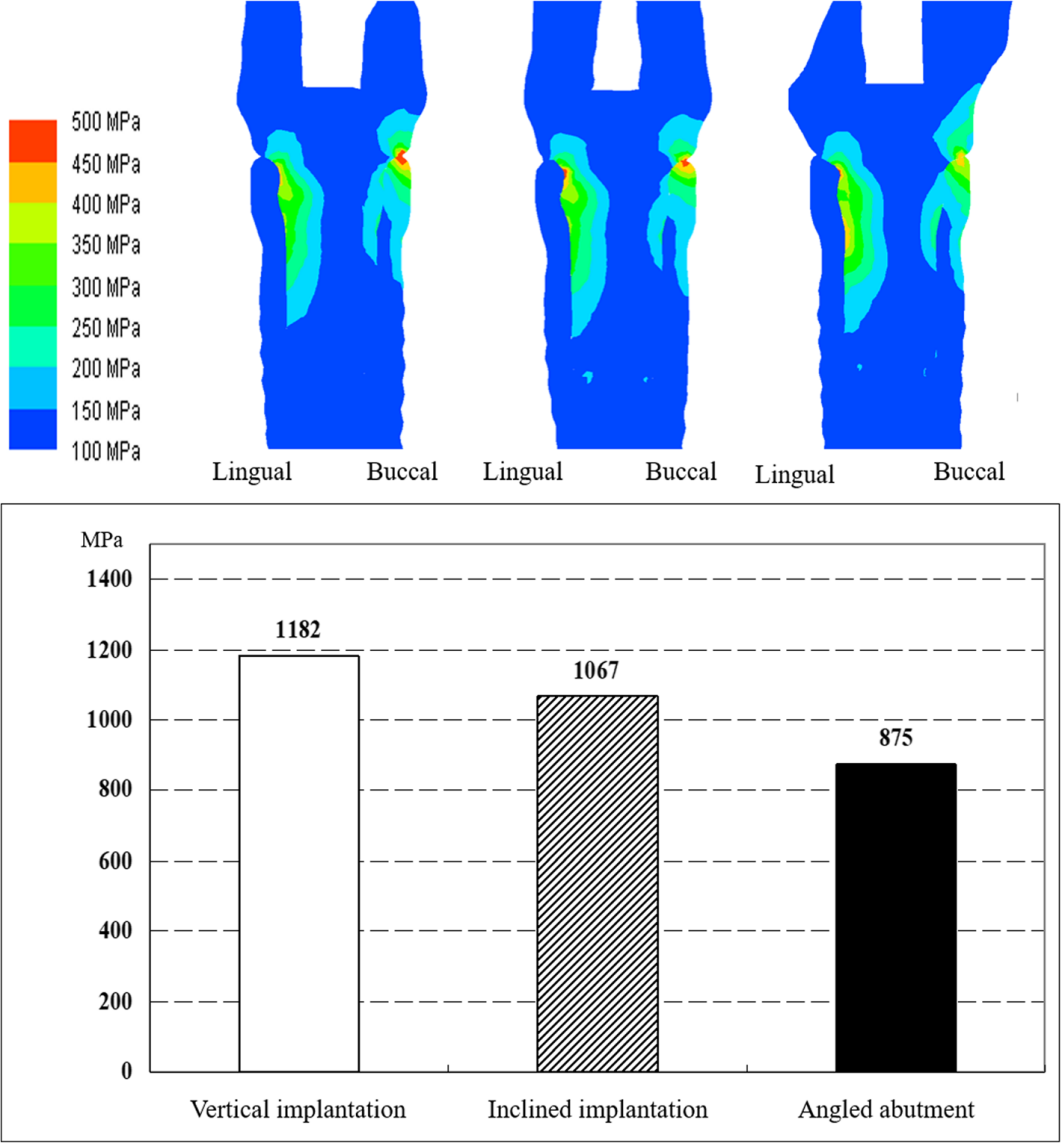
Comparison of the maximum stress under 60° loading in vertical implantation, inclined implantation, and angled abutment models

Buccal stress was concentrated on the platform area under all loading conditions, and lingual stress was concentrated at the top of the abutment tube by the occlusal force loaded near the mesiobuccal cusp tip (Figs. 16 and 17).

#### Effect of inclined implantation and angled abutment

For inclined implantation, the maximum stress was 8.4% less at a 90° load (Fig. 16) and 9.7% less at a 60° load (Fig. 17) compared with vertical implantation. With angled abutments, the maximum stress was 15.7% less at a 90° load (Fig. 16) and 30.0% less at a 60° load (Fig. 17) compared with vertical implantation.

The stress distribution map shows that the stress was dispersed more in the angled implantation model than in the vertical implantation model. The use of angled abutments was shown to disperse stress further than the inclined implantation model (Figs. 16 and 17).

Under conditions of high buccolingual deflection and increased vertical distance between the maxillary first molar and the mandibular residual ridge, the stress at the implant and abutment connection was decreased when the implant was inserted at a 20° inclination and the abutment was placed parallel to the implant, in contrast with cases in which the implant was inserted vertically and the abutment was placed parallel to the implant. Additionally, when a 20° angled abutment was used, the stress at the implant and abutment connection was decreased.

## Discussion

In this study, the positional relationship between the residual ridge and the opposing teeth obtained from actual patient data was analyzed. A detailed 3D finite element analysis was also performed to determine the ideal implant placement position and direction.

Pietrokovski and Massler reported that when a tooth is lost, the bone resorption on the buccal side is greater than on the lingual side both in the upper and lower jaws, and consequently, the dental arch shrinks [11]. They noted that when alveolar bone resorption occurs with tooth loss, the resorption on the buccal side where the cortical bone is thinner is greater than on the lingual side in both the upper and lower jaws [12].

The midpoint of the residual ridge in the first molar region after tooth extraction (the implant insertion position) was assumed to be located more on the lingual side than the natural tooth. This is consistent with the higher degree of alveolar bone resorption on the buccal side than on the lingual side. However, in relation to the center of the occlusal surface of the natural tooth, we found that the center of the mandibular ridge was widely distributed from the lingual side to the buccal side, indicating a high degree of individual difference. The reasons for this are that the site of bone resorption varies depending on the type and location of the disease causing the tooth to be extracted (such as periodontal disease or endodontic lesions); consequently, the bone shape after extraction takes various forms. Interestingly, when the adjacent teeth are missing, the horizontal deviation between the residual ridge and the opposing teeth tends to increase, possibly because the bone resorption was accelerated by the shape of the adjacent bone which was already resorbed following a previous tooth extraction. It has also been reported that resorption of the alveolar bone can be reduced by performing alveolar ridge preservation techniques on the tooth extraction fossa [13, 14]. These techniques are more effective in preventing bone resorption following tooth extractions in which the adjacent teeth are missing.

The Mechanical Finder version 6.0 software used in this study was developed to study the mechanism of femur fracture in osteoporosis patients. This software can reflect bone density obtained by computer tomography in a finite element model. It has also been reported that there are no differences in the direction of strain and the distribution of stress values between this finite element model system and a stone model with strain gauge. These findings confirm the validity of the three-dimensional finite element model in this study.

In this study, a 3.8-mm-diameter implant was selected to analyze the stress distribution between an implant inserted to existing bone and the abutment. Implant length does not affect the mechanical behavior of the abutment connection [15, 16]; therefore an 11-mm-length implant was selected. The implant and abutment were set as a contact connection (not a fixed connection), to allow for the gliding or detachment required for clinical situations. The loading point was set at a functional cusp of the mandibular molar. This point was far from the center of the implant as a result, assuming a more severe condition. The loading value in this study was set at 120 N in reference to previous studies [17, 18].

This FEM analysis showed that a large discrepancy in the buccolingual relationship increases the stress concentration at the implant/abutment connection. This tendency is amplified by the increase in the vertical dimension. Weinberg et al. [19] previously reported that the moment load through the occlusal surface increased by 15% when the buccolingual discrepancy increases by 1 mm. This load was also shown to increase by 5% when the vertical dimension increased by 1 mm in their geometric study. Generally, loosening or fracture of the abutment screw will occur when an unfavorable stress is concentrated at the implant/abutment connection [20]. Binon et al. reported that overload in the non-axial direction of the implant prosthesis provoked loosening/fracture of the abutment screw in an in vitro study [21]. The stress concentration results provided by this study are also thought to be related to prosthetic complications. Weinberg et al. also reported that the moment load increased by 30% when the cusp angle increased by 10°, which is consistent with our results. Stress concentration of the implant/abutment connection was lower in cases in which the implant placement was inclined to the opposite tooth or vertical to the occlusal plane and using an angled abutment when compared with cases with implant placement vertical to the occlusal plane in our study design. When there is an unfavorable interocclusal relationship, the following counterplans are recommended by Weinberg et al. to avoid overload to the implant prosthesis: cross-bite design, location of the implant seating surface at the center of the occlusion, use of an angled abutment, and setting of a moderate cusp angle [22]. In addition, inclined implant placement is considered to be one of the techniques used in this study to decrease the stress concentration of the implant/abutment connection by locating the implant seating surface towards the center of the occlusion. However, excessive inclination is thought to cause lateral overload and to have a negative influence on the implant/bone contact [23, 24]. The use of angled abutments was more effective in decreasing the stress concentration in this FEM study, most likely because the abutment head is close to the occlusal surface (loading point). Additionally, the different material properties between the crown and abutment and the hollow structure of the abutment screw space also contributed to this result. Therefore, if there is a buccolingual discrepancy and a large vertical dimension, the use of an angled abutment is thought to be the most advantageous method.

There were several limitations in this study. The age of the participants in experiment 1 was relatively high, so our results could not be considered universal for all generations. In addition, the reason of tooth extraction in each patient could not be recorded in this study because it was a cross-sectional study; therefore, causality of bone resorption and missing duration is unclear. Meanwhile, the tendency of buccolingual discrepancy between the residual ridge and the opposing tooth is similar to previous studies [11, 12], and also, this study focused on the degree of vertical distance due to tooth extraction. These results are thought to be helpful for perceiving the three-dimensional bone change after tooth extraction. Future longitudinal studies are required that follow cases from before extraction to after implant placement. FEMs in experiments 2 and 3 are not also universal for all clinical cases. In this study, the model of this FEM came from a 53-year-old female’s CT scan data having a typical absorbed bone shape. However, using the patient CT scan data for FEM model is considered to be meaningful compared to artificial models. In addition, this study focused on the distribution of the stress between implant and abutment junction under the presence of horizontal and vertical discrepancy and the effect of inclined implantation or using angled abutment. In this model, the remaining bone width at buccolingual aspect was enough; therefore, the impact of the bone shape is considered to be small, and the results of this study are thought to be meaningful.

## Conclusion

In cases of progressive alveolar resorption with a large clearance between the implant and the opposing teeth, a higher stress concentration was observed at the joint between the implant and the abutment. Our findings also showed that stress concentration around this area can be reduced by the use of inclined implantation and angled abutments under the condition of a horizontal offset between the implant and opposing teeth.

## Data Availability

The datasets used during the current study are available from the corresponding author on reasonable request.
